# The importance of being apt: metaphor comprehension in Alzheimer's disease

**DOI:** 10.3389/fnhum.2014.00973

**Published:** 2014-12-02

**Authors:** Carlos Roncero, Roberto G. de Almeida

**Affiliations:** Department of Psychology, Concordia UniversityMontreal, QC, Canada

**Keywords:** metaphor, Alzheimer's disease, figurative language, language comprehension, aptness, familiarity, abstraction, simile

## Abstract

We investigated the effect of aptness in the comprehension of copular metaphors (e.g., *Lawyers are sharks*) by Alzheimer's Disease (AD) patients. Aptness is the extent to which the vehicle (e.g., *shark*) captures salient properties of the topic (e.g., *lawyers*). A group of AD patients provided interpretations for metaphors that varied both in aptness and familiarity. Compared to healthy controls, AD patients produced worse interpretations, but interpretation ability was related to a metaphor's aptness rather than to its familiarity level, and patients with superior abstraction ability produced better interpretations. Therefore, the ability to construct figurative interpretations for metaphors is not always diminished in AD patients nor is it dependent only on the novelty level of the expression. We show that Alzheimer's patients' capacity to build figurative interpretations for metaphors is related to both item variables, such as aptness, and participant variables, such as abstraction ability.

## Introduction

Why are we so good at understanding metaphors when they express such obvious falsities? Upon hearing *Juliet is the sun*, how should we interpret Romeo's state of mind? Clearly, what he intends to express about Juliet (the *topic*) seems to be easily understood by attributing to her some property of the sun (the *vehicle*)—perhaps that of sheer brightness, uniqueness, or being vital for life. Although copular metaphors—those with the form *x is y*—are pervasive in natural language and explored profusely in literary works, their comprehension might require considerable cognitive effort. This effort may come from different levels of analysis that metaphors call for, including computing the literally anomalous meaning (what is *said*), interpreting properties of topic and vehicle, and arriving at an interpretation that is assumed to be close to what the speaker *intended* to express. Understanding a metaphor, thus, may engage many systems—from linguistic parsing and semantic composition to executive functions involved in attaining an interpretation that goes beyond what the sentence expresses literally.

We report a study on the interpretation of metaphors by patients diagnosed with probable Alzheimer's disease (AD). Considering the well-documented difficulties that AD patients have with linguistic processes (e.g., Manouilidou et al., 2009), semantic memory (e.g., Whatmough and Chertkow, 2002; Capitani et al., 2003), and working memory, in particular with executive functions (e.g., Baddeley et al., 1986; Bäckman et al., 2005), the task of interpreting non-literal sentences might seem a daunting one for this population. Surprisingly, however, only four studies to our knowledge have investigated how AD patients interpret metaphors (Winner and Gardner, 1977; Papagno, 2001; Amanzio et al., 2008; Maki et al., 2012). These studies differ substantially in method, language, types of metaphors employed, and stimulus properties. Only the study by Amanzio et al. (2008), for example, controlled for level of conventionality, contrasting conventional and familiar metaphors with novel ones. They found that AD patients have difficulty with novel metaphors, but their comprehension of conventional metaphors was similar to that of healthy controls. They suggested that the main reason for the novel-metaphor impairment in AD might be defective executive functions and what they called “verbal reasoning,” which are deemed necessary to compute relations between novel topic-vehicle combinations. Conventional metaphors, in contrast, were argued to rely less on executive functions and more on retrieving an associated meaning from semantic memory^1^.

In the present study, we investigate the role of another variable in metaphor interpretation by AD patients: *aptness*. This variable reflects the degree to which properties of the vehicle capture properties that are applicable to (or can be predicated about) the topic. For example, in *Lawyers are sharks* the vehicle *shark* by hypothesis activates properties that might be true of *lawyer*. Crucially, aptness is independent of conventionality and familiarity: an unfamiliar metaphor can still be apt based on the properties of the vehicle that are applicable to the topic; and a conventional metaphor can be inapt if the common figurative meaning of the vehicle does not apply to the topic. We also evaluated to what degree a patients' ability to perform abstractions could predict metaphor interpretation—on the assumption that abstraction might be required to detach the literal meaning from the expression and generate an interpretation that approaches the intended meaning.

We start off with a brief discussion on the comprehension of different types of figurative expressions in AD: proverbs, sarcasm, idioms, and metaphors. Our main goal is to gather the pattern of performance of AD patients in diverse types of tests employed in the investigation of figurative language, and which motivate our study on metaphor, reported below. A secondary goal of our discussion on figurative language in AD involves evaluating both subject and item variables employed in these studies, which is crucial for understanding how a meaning that approximates that of the intended message is attained and how it may be disrupted in AD.

## Figurative language in alzheimer's disease

Thus far, 22 studies have investigated diverse forms of figurative language in AD—including proverbs, idioms, sarcastic expressions, and metaphors (for a recent review, see Rapp and Wild, 2011)^2^. What seems to be common to these forms of expression is that there is a stark contrast between what is *said* and what is *intended* by a token utterance. We follow here a classical distinction in pragmatics (e.g., Grice, 1989) assuming that what is *said* is the literal interpretation of the expression, its compositional meaning based on word meanings and how they combine structurally.^3^ We thus take what is *intended* by a given expression to be what is implicated (rather than explicated), or what the speaker intends to express, whether this intention can be easily calculated (such as the ironic *It is hot in here*, uttered by a visitor to Yukon in January) or not (*Juliet is the sun*). While this distinction has been well established in many research circles in cognitive science, what more recent psycholinguistic and cognitive neuroscience research have shown is that numerous variables play an important role in the process of calculating the intended message from what is said (see, e.g., the papers in Gibbs, 2008, and Roncero and de Almeida, 2014, for reviews). The main variables of interest include the expression's familiarity (Blasko and Brihl, 1997; Thibodeau and Durgin, 2011), conventionality (Bowdle and Gentner, 2005; Gentner and Bowdle, 2008), and aptness (Chiappe and Kennedy, 1999; Jones and Estes, 2005, 2006; Glucksberg and Haught, 2006). In addition, in the more specific case of figurative expressions in AD, variables such as the degree to which the patient must rely on executive functions such as inhibition and abstraction (Laflache and Albert, 1995; Chapman et al., 1997; Papagno et al., 2003), and whether or not the expression is “frozen,” i.e., stored as a whole (Amanzio et al., 2008; Rassiga et al., 2009), have been investigated. We will take this last variable as the perspective from which we discuss briefly the studies on AD patients' interpretation of figurative expressions. The main reason for focusing on this variable is that “frozen” and “non-frozen” expressions by hypothesis rely upon different cognitive resources. In frozen expressions (e.g., idioms), the nonliteral meaning is fixed in the sense that the interpretation relies more on the retrieval of a conventional meaning from semantic memory than on the computation of a novel meaning. In contrast, non-frozen expressions, such as in most metaphors, actual interpretation requires the computation of a meaning, rather than retrieval from memory: even in the case of familiar and conventional metaphors, the actual property retrieved from the vehicle to predicate on the topic is flexible because numerous properties are usually associated with a given conventional vehicle (e.g., *ruthless, aggressive, sneaky*, etc., for *shark*; see Roncero and de Almeida, 2014)^4^.

### Proverbs

A proverb often involves the “teaching of a lesson”—which reflects its *intended* message. For example, *Too many cooks spoil the broth* suggests that too many people involved in a single project can spoil the end result. Although one could argue that these expressions are compositional, for their literal meanings are obtained from their constituents, proverbs are used to express something else, perhaps analogous to the expression itself—and thus they require the retrieval or the computation of another message. Studies have shown that AD patients prefer literal (or “concrete”) rather than figurative (“abstract”) interpretations of proverbs (e.g., *Rome wasn't built in a day*; Code and Lodge, 1987) and familiar, proverb-like sentences (e.g., *He's saving up for a rainy day*; Kempler et al., 1988). These results have been obtained with both, free-interpretation (Code and Lodge, 1987) and multiple choice tasks (Kempler et al., 1988), suggesting that AD patients' abstracting abilities might be impaired, making it difficult for patients to go beyond what is explicitly said in the sentence. However, Brundage (1996) found that the difficulty with proverbs is mostly due to their familiarity, suggesting instead that comprehension of proverbs relies more on remembering an associated meaning, which becomes stronger with increased familiarity, rather than relying on a process of abstraction from the words in the proverbs. Consistent with these results, Laflache and Albert (1995) found AD patients could provide accurate proverb interpretations, despite showing impaired abstract thinking abilities. This effect was further confirmed by Chapman et al. (1997), but only for familiar proverbs, with patients showing an impairment for unfamiliar proverbs. Chapman et al. also found that when patients were given a multiple-choice task with four alternatives, including “concrete” and “abstract” (i.e., figurative) interpretations, the effect of familiarity disappeared, with patients having difficulty selecting the figurative interpretation. Therefore, the different conditions (multiple-choice vs. verbal explanation) appear to have distinct cognitive demands, as AD patients performed worse in the multiple-choice condition.

### Sarcasm

Sarcasm is a form of expression that usually stands in opposition to what is said: *It is hot in here* (Yukon, circa January) means “it is cold” (or, more properly, its negation: “not hot”). While the meaning of the expression itself is compositional, its intended meaning needs to be inferred from a given intonation or context. The investigation of sarcasm interpretation in AD has also employed either free-interpretation (Kipps et al., 2009; Rankin et al., 2009) or multiple-choice (Maki et al., 2012) tasks. Both Kipps et al. (2009) and Rankin et al. (2009) employed two subtests of the TASIT (The awareness of social interference test; see McDonald et al., 2003) in which patients watched vignettes with actors engaging in dialogs that could be interpreted as being either sincere or sarcastic (e.g., *Ruth*: *Great movie, wasn't it?* […] *Michael*: […] *I feel I could see it another dozen times*). The dialogs contained the same sentences, thus, participants had to rely on extra-linguistic cues such as intonation, facial expressions, and gestures to judge whether the actors in the scene were being sincere or sarcastic. Both studies found no impoverished sarcasm comprehension in AD patients compared to controls. In the study by Maki et al. (2012), however, AD patients did perform significantly worse than both, a group of healthy elderly controls and a group of patients with mild cognitive impairment (MCI): in fact the AD patients chose the literal interpretation significantly more than other control groups. As with proverbs, the pattern of patients' performance may be due to the task: the use of a multiple-choice paradigm rather than interpretations through verbal explanations.

### Idioms

Idioms such as *pushing up daisies* (meaning “dead and buried”) and *hit the sack* (“going to bed”), represent meanings that are not compositional and might require retrieving the associated meaning from memory. Studies investigating idioms in AD have also found that performance in verbal descriptions is superior to that in multiple-choice of picture selection tasks (Papagno et al., 2003). However, Papagno et al. (2003) also showed that performance in the picture selection task varies with the nature of the pictures presented as alternatives. When patients are presented with pictures representing literal and figurative interpretations of common Italian proverbs (e.g., *to have a green thumb*), they perform at chance (e.g., selecting either a picture of a man with a thumb full of green paint or a picture of a woman gardening), but when the alternative pictures represent either the figurative meaning (the gardener) or an unrelated picture containing the depiction of one word referent from the idiom (e.g., someone with a thumb stuck at a drawer), patients select the figurative interpretation significantly more than the alternative. These results suggest that AD patients are capable of interpreting figurative expressions, but have difficulty efficiently suppressing the literal interpretation when it is presented as an alternative (Papagno et al., 2003).

The hypothesis that AD patients have difficulty suppressing a literal interpretation was further investigated in two matching tasks by Rassiga et al. (2009). In the first, patients had to choose among four drawings the one that corresponded to the interpretation of the idiom. In the second, patients had to match the idiom to one of four alternative words, one associated with the figurative interpretation, one associated with a word in the idiom (the literal alternative), and two unrelated words. Performance was worse than controls in both conditions. In the picture-matching task, participants chose more often the picture corresponding to the literal than to the figurative interpretation. In the sentence-to-word task, however, patients chose the word representing the figurative meaning of the idiom significantly more than other alternatives. Rassiga et al. found that performance on the sentence-to-word task was predicted only by executive-function scores, as measured by a dual-task that included digit span and paper-and-pencil maze tasks (Baddeley et al., 1997). These results again suggest that difficulty in idiom interpretation might be due to failure of inhibition of the literal interpretation, while the degree of inhibition needed can be affected by test modality: picture matching requires more inhibition than single-word matching possibly because alternative scenes involve more working-memory resources to match to an appropriate sentence. By extension, verbal explanations would have required even less inhibition as they do not involve foils, although Rassiga et al. (2009) did not employ this technique. Indeed, in studies in which AD patients were asked to provide verbal explanations for idioms (Papagno, 2001; Amanzio et al., 2008), no impairment was found.

### Metaphors

Copular metaphors, in contrast to idioms, require identifying the relevant property associated with the vehicle that can be applied to the topic (Ortony, 1979; de Almeida et al., 2010). Thus, in *Juliet is the sun* one needs to search for possible ways in which the topic (*Juliet*) could be predicated by the vehicle (*sun*). As suggested by Papagno (2001, p. 1458), metaphors involve “an active search of the specific semantic attribute,” more so than other types of figurative language. Because AD patients have impaired executive functions (Baddeley et al., 1986), it follows that metaphors' increased cognitive demands may cause interpretation difficulties, in particular in the search for an appropriate intended meaning.

In what was perhaps the first study examining metaphor interpretation in AD, Winner and Gardner (1977) asked seven individuals (all diagnosed with pre-senile dementia and probable AD) to select, among four pictures, the one that best matched a given metaphorical statement (e.g., *a heavy heart can really make a difference*). Two of the picture-types used are relevant to the present paper: one that matched the figurative meaning of the metaphor (a picture of a man crying), and a second one, which displayed the literal form (a person having difficulty walking due to carrying a large red heart). AD patients were found to pick the picture representing the metaphorical meaning as many times as the picture representing the literal meaning (45 and 44% respectively). This result is consistent with those found for proverbs, idioms, and sarcasm, which show that AD patients have difficulty selecting the *intended* meaning in the presence of a literal competitor^5^.

Papagno (2001), however, employed a verbal explanations task to examine AD patients' comprehension of idioms and metaphors over a 6-month period. Both the idioms and metaphors were considered highly familiar in Italian, to the extent that their meanings could be found in a dictionary. The assumption was that AD patients should have known these expressions, but could have “lost” them during the disease progression. At first examination, only four patients demonstrated impairment for nonliteral language, with metaphor comprehension being the *least* impaired linguistic ability. Among the errors produced, however, a distinction did emerge between idioms and metaphors. Whereas the most common error for idioms was a literal interpretation, the most common error for metaphors was an inability to produce a response. When AD patients were retested at a later stage, there was an overall decrease in nonliteral language comprehension, yet further analysis showed this result was attributable to metaphors only. AD patients showed no decrement for idioms. These results led Papagno to suggest that language impairment, especially for figurative language, is not an early symptom of AD and may only occur late into the progression of the disease.

In addition to using verbal explanations rather than a matching paradigm, Papagno's (2001) study contrasts with Winner and Gardner's (1977) for the familiarity of the items used. Winner and Gardner (1977) did not report the familiarity level of their items, whereas Papagno's metaphors were chosen for their high familiarity. It is possible that the distinct results in the two studies reflect the familiarity level of the individual items in the study. In other words, comprehension was better in Papagno's study because the metaphors were more familiar than those used by Winner and Gardner. This hypothesis was investigated by Amanzio et al. (2008), who compared AD patients' interpretation of novel metaphors with conventional ones—the same conventional metaphors used by Papagno (2001).

Amanzio et al. (2008) predicted that AD patients would show good interpretation for conventional metaphors, whose meanings are well known, because patients would simply need to retrieve the associated figurative meaning from memory, as done for idioms. In contrast, AD patients would have more difficulty with novel metaphors, whose figurative interpretation must be constructed based on possible relationships between topics and vehicles. Thus, the assumption was that for novel metaphors there were no figurative meanings stored in memory. To further support this retrieval-construction dichotomy, Amanzio et al. also compared participants' ability to interpret new and conventional metaphors to patients' interpretations for idioms as these are also assumed to simply rely on memory retrieval. Thus, patients and controls were predicted to show similar performance for idioms and conventional metaphors that rely on retrieval, but worse interpretation for novel metaphors whose meaning needs to be constructed. The results supported their predictions. In addition to conventional metaphors, AD patients also showed good interpretation (similar to controls) for idioms. Novel metaphors were the only category where AD patients displayed a relative impairment compared to controls. AD patients' performance in verbal, visual reasoning, and executive-function tasks were also the best predictors of metaphor interpretation scores. Amanzio et al. took these results to support their hypothesis that the main obstacle faced by individuals when interpreting novel metaphors is the need to construct a meaning due to impaired executive functions: “if the central executive is damaged, the ability to create a new resemblance, required to understand a novel metaphor, may be defective” (p. 7). When the comprehension process relies on retrieval rather than construction, AD patients' verbal explanations is similar to controls', as observed for idioms and conventional metaphors. However, when the process relies on construction, comprehension can be impaired, as observed for novel metaphors.

## Study 1: the comprehension of metaphors in alzheimer's disease

We set out to study metaphor comprehension in AD with three main goals in mind. First we were interested in understanding how the possible breakdown of metaphor comprehension in this population might inform us about the normal processes involved in metaphor processing. We see the investigation of patterns of impaired performance—both in groups of patients and in single-case studies—as an important method for understanding how unimpaired linguistic and cognitive systems work (see Caramazza, 1986; Zurif et al., 1989). Clearly, the contrast between meaning construction and meaning retrieval suggested by studies on figurative language with AD patients implies that different cognitive mechanisms might be recruited in metaphor comprehension, and that empirical results depend on task and stimulus variables. Thus, a second goal in our study was to explore the role of different variables underlying metaphor comprehension and particularly the role of familiarity and aptness. And finally, our third goal was to understand figurative language comprehension in AD proper, and more specifically how the semantic and pragmatic systems might breakdown with the disease. The paucity of metaphor comprehension studies in AD is surprising given how productive these expressions are in natural language. An exploration of how metaphors are understood can ultimately help us understand how linguistic, semantic memory and working memory systems are affected with the progression of the disease.

### Familiarity and aptness

As we have seen in our brief review of the literature on figurative language in AD, comprehension is better for what we referred to as “frozen” than for “non-frozen” expressions—and this difference reflects distinct cognitive demands. Whereas frozen expressions require retrieving an associated meaning, non-frozen expressions, such as most copular metaphors, require constructing a meaning based on the relation between topic and vehicle words. Whether or not good performance is observed, however, is related to two additional factors: task modality and familiarity. Patients typically perform better when asked to provide verbal explanations rather than when they are asked to match an expression to a picture, word, or sentence alternative; and they also perform better when expressions are familiar. Familiarity, however, interacts with other ways of conceiving how one attains the meaning of a figurative expression. One of them is what Giora (1997) called *saliency*. She argues that both the literal and figurative meanings, when available, compete during comprehension, but the meaning with the highest level of *saliency* will be chosen. Saliency, then is akin to the activation level that one meaning will reach, winning out against a competitor, regardless whether the winner is a literal or a figurative interpretation. With regards to a particular figurative expression, then, the greater the familiarity, the more strongly that expression will be associated with a nonliteral meaning. In other words, familiarity has the effect of making the nonliteral meaning more salient, which makes subsequent retrieval of those meanings easier. Supporting this argument, studies with healthy adults found that familiar nonliteral expressions are read faster than less familiar ones (e.g., Blasko and Brihl, 1997).

The impact of familiarity on saliency, then, is somewhat straightforward: increased experience leads to stronger traces in semantic memory. Gentner and Bowdle (2008), for example, argue that vehicles initially have only an associated literal meaning, but can gain an additional meaning from its frequent use with different topics. Over time, exposure to the nonliteral use of the vehicle leads it to become stored in semantic memory and thus retrieved whenever the vehicle is heard or read in a statement. A metaphor such as *That film was a blockbuster* is taken to mean that the film had great commercial success, as opposed to mean that the film exploded a city block. Gentner and Bowdle refer to such vehicles as *conventional*. In the case of *dead metaphors*, vehicles have become so conventional that only a nonliteral meaning remains. It is worth noting that these highly conventional vehicles were the types used by Papagno (2001) and Amanzio et al. (2008). They found that AD patients interpreted conventional metaphors as well as healthy controls, but were worse than controls when asked to interpret novel metaphors.

In contrast to familiarity, aptness is not related to one's experience but it is rather more related to the salient properties activated by the expressions' topic and vehicle. More specifically, aptness is seen as reflecting the degree to which the vehicle term captures salient properties of the topic (McCabe, 1983; Chiappe et al., 2003b); thus, an expression is more apt when the vehicle captures many properties of the topic. For example, the word *rail* is not a conventional vehicle (Jones and Estes, 2006) and lacks a strongly associated nonliteral meaning. Thus, the statement *John is a rail* has no clear meaning other than the anomalous literal one. Pairing the vehicle with the topic *fashion model*, however, to state *That fashion model is a rail* conveys that the person is extremely thin and skinny—like a rail. Here, the expression is interpretable not from experience with the vehicle, but rather because the statement is highly apt: the vehicle *rail* captures salient attributes associated with *fashion model* (i.e., thinness).

Aptness has been found to correlate strongly with ease of comprehension (Chiappe et al., 2003a). Some researchers (e.g., Glucksberg, 2003, 2008) even argue that aptness is a more important variable than familiarity for metaphor comprehension because unfamiliar metaphors can be well understood if the statements are sufficiently apt (e.g., Glucksberg and Haught, 2006). For AD patients, aptness could make comprehension easier because the relevant properties are salient for both the topic and the vehicle. Patients would then be biased toward selecting those properties as the ones needed for interpretation because they have the highest saliency level. For this reason, statements such as *The senator is a fossil* are more apt and easier to understand than *The track star is a fossil*. Although fossil in both cases has the relevant attribute of *old*, this attribute is more salient of senators than track stars, which makes it easier to employ the relevant attribute (Jones and Estes, 2005, 2006).

In summary, it can be argued that both familiarity and aptness are variables that might account for better metaphor comprehension. It has been difficult, however, to determine which of these variables—aptness or familiarity—is more important because several studies report significant positive correlations between participants' aptness and familiarity ratings (e.g., Jones and Estes, 2006; Thibodeau and Durgin, 2011). Such results have cast doubt on studies that have relied on subjective ratings of familiarity from participants because these ratings may have actually reflected the items' aptness level (Jones and Estes, 2005). To remedy this problem in the present study, we used instead an objective measure of familiarity in our analysis: Internet frequency counts, gathered using the guidelines from Roncero et al. (2006). These counts were first used to reduce an initial large cohort of metaphors to those used in the present study. For aptness judgments, we collected norms from a large group of older adults. By using two different types of measurements (subjective ratings for aptness, but objective ratings for familiarity), we aimed at better tapping into these distinct variables for metaphors whose meanings were absent from a dictionary.

### Other cognitive and linguistic variables

In addition to examining how different levels of familiarity and aptness impact metaphor interpretation, we also examined whether a participant's ability to infer a relationship between two objects would predict their ability to interpret metaphors. Recall that constructing the meaning of a metaphor (e.g., *time is money*) often involves understanding the relationship between two terms (e.g., *time-money*). The vehicle (*money*) is understood to predicate something about the topic (*time*), and interpretation requires understanding what properties about *time* are being made salient by *money* (e.g., that time is valuable). Therefore, we were especially interested in scores obtained in the Similarities subtest of the Wechsler Adult Intelligence Scale (WAIS-IV). In this task, participants are asked how two objects are “alike” (e.g., *horse-tiger, food-gasoline*) with different scores allocated based on the quality of the answer provided. This score is a good measure of a participant's ability to create new relations between two objects, and numerous studies have used it to assess AD patients' executive functions and in particular abstracting abilities (e.g., Laflache and Albert, 1995; Chapman et al., 1997; Helmes and Ostbye, 2002). Our prediction was that the best interpretations would be produced by patients who demonstrate the greatest ability to list salient similarities between two objects.

We also examined working memory as measured by the digit span task (also a subtest of WAIS-IV). Because constructing the meaning of a metaphor presumably requires being able to hold both the topic and vehicle terms in working memory, participants with an extremely short digit span could have difficulty holding the topic term in working memory once the vehicle term itself is processed. Consequently, participants with a very limited digit span would be expected to display poor metaphor interpretation abilities. Finally, we examined if the form of the expression would impact interpretation. More specifically, we tested whether or not participants would have less difficulty interpreting a metaphor such as *The mall is a zoo* if it was presented as a simile (*The mall is like a zoo*). Career of Metaphor theory (Bowdle and Gentner, 2005; Gentner and Bowdle, 2008) proposes that the comprehension of a novel metaphor involves a comparison process between topic and vehicle (e.g., *teachers-sculptors*). More specifically, to understand a new metaphor such as *Teachers are sculptors*, the metaphor must be understood as a simile via the form *Teachers are like sculptors*. If this is the case, when AD patients are presented with a novel metaphor, they might attempt to mentally transform it into a simile to compare topic and vehicle. However, a central executive impairment (Baddeley et al., 2001; Amanzio et al., 2008) might hinder the ability to perform such a metaphor-to-simile conversion. In order to test this hypothesis we asked participants to interpret both metaphors and comparable similes, which enabled us to determine if interpretation was better when the metaphors were presented directly as similes.

In summary, in order to control for the possibility that our familiarity ratings would reflect the item's aptness rather than its general frequency, we collected Internet frequency counts as an objective measure of the items familiarity. We predicted that apt metaphors would be better understood, regardless their familiarity level, but this effect would interact with participants' abstraction ability. More specifically, our prediction was that patients with higher similarity scores would produce better interpretations. Lastly, we examined whether AD patients would understand the relationship between topic and vehicle better when these were presented as similes rather than metaphors.

### Methods

#### Participants

Eleven patients with probable AD (age range 55–86), diagnosed with mild-to-moderate cognitive impairment were recruited with assistance from the Alzheimer's Society of Montreal, as well as Sunrise of Beaconsfield, a retirement community in Beaconsfield, Quebec. Participants were referred to us as individuals who had been given a diagnosis of AD according to the criteria specified by the National Institute of Neurological and Communicative Disorders and Stroke-Alzheimer's Disease and Related Disorders Association (NINCDS-ADRDA; McKhann et al., 1984), and had no other diagnosed dementia or pathology. We also examined patient files to verify the diagnosis. The study was fully explained to each participant and they gave written informed consent to participate in the study (the protocol was approved by the Institutional Review Board of the Douglas Mental Health University Institute). MoCA (Montreal Cognitive Assessment; Nasreddine et al., 2005) and MMSE (Mini-Mental State Examination; Folstein et al., 1975) were administered to all AD patients. Further criteria for participating in the study was patients' ability to understand and follow commands, and have an MMSE score of at least 16. Demographic and neuropsychological data for all participants appear in Table 1.

**Table 1:** **Demographic and neuropsychology data for AD patients and normal controls**.

**Participant**	**Age**	**Education (years)**	**MoCA**	**MMSE**	**Digit span**	**Similarities**
AD1	84	17	19	26	10	13
AD2	68	14	21	29	14	29
AD3	82	15	26	29	14	29
AD4	55	15	14	16	8	21
AD5	71	14	16	23	10	23
AD6	83	17	22	30	12	21
AD7	84	21	17	26	13	11
AD8	76	13	14	21	13	0
AD9	86	11	16	18	13	19
AD10	81	11	13	24	8	23
AD11	71	12	04	16	8	0
*Mean*	76.5	14.5	16.6	23.5	11.2	17.2
(*SD*)	(9.44)	(2.98)	(5.73)	(5.15)	(2.44)	(10.13)
NC1	63	16	28		12	23
NC2	76	15	26		12	27
NC3	65	18	29		13	25
NC4	73	12	28		10	26
NC5	65	15	27		12	29
NC6	86	16	29		11	28
NC7	75	15	29		13	25
NC8	69	15	28		12	28
NC9	70	06	28		11	26
NC10	81	14	28		10	25
*Mean*	72.3	14.2	28		11.6	26.2
(*SD*)	(7.41)	(3.25)	(0.94)		(1.07)	(1.81)

*AD, Alzheimer's Disease; NC, Normal Controls; MoCA, Montreal Cognitive Assessment (Nasreddine et al., 2005); MMSE, Mini-Mental State Examination (Folstein et al., 1975); Digit Span and Similarities, Subtests of the WAIS-IV; SD, Standard Deviation*.

Ten healthy elderly controls, with an age range of 63–86, were recruited from Sunrise of Beaconsfield, were caregivers of the participants diagnosed with AD, or were recruited from the general public. For controls, only the MoCA was administered, with the requirement that all controls obtain a score above 25. All participants (AD patients and controls) were native speakers of English, or were bilinguals with a fluent command of English (case of two individuals), having attended university in English and worked professionally their entire lives in English. Therefore, these participants' English proficiency level was considered sufficient for the present study. All participants had a minimum education level of 6 years (Table 1).

#### Materials

The preparation of the stimuli involved two main phases. The first included an aptness-rating task, with a group of healthy elderly individuals, and the collection of frequency counts from the Internet, using the Google Search Engine (see below). These frequency counts allowed us to first identify those metaphors that were familiar and those that were unfamiliar. In the second phase, interpretation norms for a subset of metaphors from the first phase were obtained with another group of healthy elderly individuals. We also collected frequency counts from the Corpus of Contemporary American English (COCA; Davies, 2009) for the metaphors presented to participants. These COCA scores allowed us to check that the Internet counts for the metaphors do accurately reflect general frequency and served as a second objective rating of familiarity. Aptness, familiarity, and interpretation norms for the materials employed in the present study appear in the Supplementary Material.

***Aptness***. Twenty healthy elderly controls (age range 60–83; 10 females), all native speakers of English, were recruited from the general public and given monetary compensation for completing a rating task. These participants did not take part in the subsequent metaphor interpretation task. They were presented a booklet containing 84 metaphors (e.g., *Trees are umbrellas*) taken from another study (Roncero and de Almeida, 2014). Below each expression, there was a scale ranging from 1 to 7. Participants were asked to rate how apt they found each metaphor, where 1 was labeled *not apt*, 4 as *moderately apt*, and 7 as *very apt*. Aptness was explained as how valid they thought each statement was. *Politics is a jungle* was given as an example of an apt statement, while *Politics is a beach* was given as an example of a less apt statement.

***Familiarity***. The Google search engine was used to collect Internet frequency counts following the guidelines set by Roncero et al. (2006). In this method, a metaphor (e.g., *Music is medicine*) is written within quotation marks into the search box to produce a list of websites where the searched item was found. The first website listed is examined, and in general, if the metaphor is used in a figurative manner and expressing the meaning of the searched metaphor, then its production is included in the frequency counts. The next website listed is then examined, and so on, until a cut-off point of 30 “hits” is reached. To be clear, more than simply the first 30 websites are examined. Websites are examined one-by-one until a maximum of 30 productions that properly express the meaning of the searched metaphor is found. Furthermore, repetitions of the same metaphor within a website (e.g., when posts quote the same sentence repeatedly), and repetitions of the same title for a song or book across websites, are counted only once within the total; consequently, this method is more meticulous than simply examining the first 30 websites listed. Regarding the cut-off, the high number of websites listed by Google for certain metaphors can be greater than 10,000 for less familiar metaphors such as *Cities are jungles*, or near the millions for very familiar metaphors such as *Time is money*. As a practical solution, Roncero et al. chose 30 after remarking that few metaphors actually yield this number of productions. Expressions that reach a familiarity score of 30 would also still reflect a relatively higher level of familiarity compared to the rest of the expressions.

Note that the number of hits that Google initially lists is separate from the list of websites it lists. For example, although Google may inform that there are 11,300 hits for the metaphor *Cities are jungles*, the number of websites initially listed is only 99. After listing these websites, Google will print the statement “in order to show you the most relevant entries, we have omitted some entries very similar.” This number varies per expression; for example, while it is 99 results prior to the Google statement for *Cities are jungles*, it is 243 for *Lawyers are sharks*, and the non-listed hits come from the same websites that Google already listed. In the present study, we checked all websites until a frequency count of 30 was reached or when Google printed the statement “in order to show you the most relevant results…” for the searched metaphor. Therefore, a frequency count for a metaphor based on the Google search engine reflects how many distinct websites displayed a spontaneous use of the expression.

To further ensure that these Internet frequency counts were tapping into expression familiarity, we also collected COCA frequency counts for the metaphors that were employed in the interpretation task. However, for these frequencies a less restrictive set of guidelines was used, and no maximum cut-off points were applied. The topic (e.g., *time)* was entered as the search word and the vehicle (e.g., *money*) was entered as a collocation within five words before or after the topic. Each listed production was then checked for whether it was expressing a literal or figurative interpretation. For example, *He was run out of both time and money* would be considered a literal interpretation because it refers to time and money in a concrete, non-figurative, manner. In contrast, a sentence such as *He didn't understand that for his lawyer time was money* would be included because it reflects the figurative meaning of *time is money*. We also included examples in the overall count when the exact structure was different, but the expressed meaning was the same. For example, while participants in the present study interpreted *Time is money*, the COCA count totals included productions such as *Time was money, money is equivalent to time*, and *In this profession, money and time are equivalent*. In summary, a given COCA example was excluded from the count totals only when it expressed a literal interpretation of the topic-vehicle relationship.

***Selection of metaphors***. Prior to the study, we decided to employ a set of metaphors that varied in terms of aptness and familiarity, and that only 20 expressions would be presented to each AD patient because a larger number of items would conceivably cause fatigue, due also to other pre-experimental tasks involved. We identified 5 metaphors (or their equivalent similes) that were apt but not familiar (aptness rating higher than 3.5, but an Internet frequency count less than 15), and 5 metaphors that were neither apt nor familiar (an Internet frequency count less than 15, and an aptness rating less than 3.5) There were no items with an Internet frequency count greater than 15 that had an aptness rating less than 3.5; these metaphors would have been categorized as familiar, but not apt. Therefore, to complete our cohort, we identified 10 metaphors that were apt and familiar (an Internet frequency count greater than 15 and an aptness rating higher than 3.5). However, as we later discuss, two of the apt and familiar metaphors were ultimately removed from the analyses due to difficulty in interpretation.

***Interpretation norms***. In order to collect interpretation norms—i.e., to obtain the most common interpretation for each expression—a booklet containing the 20 selected metaphors was created. This booklet was presented to 20 healthy controls (age range 60–84; 14 females) that had not participated in the ratings norms nor served as controls in the subsequent interpretation task. In this booklet, each metaphor was presented within a sentence that asked participants to state which property was being expressed about the topic (e.g., *Education is a stairway because education is…*). This method helped facilitate answers that would reflect a particular property or adjective. People were asked to write their answers on a line placed beneath each expression.

The different properties expressed by each participant were collapsed under a single property when they were considered synonyms. For example, *ruthless* and *aggressive* for *Lawyers are sharks* were grouped together under the property label *ruthless*, while *valuable* and *important* for *Time is money* were both categorized under the property *important*. Participants also sometimes wrote elaborate sentences that had the similar meaning of a particular property, without necessarily using a synonym of that particular property. For example, one participant wrote *Lawyers are sharks because lawyers are out to get you!* This sentence was categorized as expressing the property label *ruthless* for *lawyers are sharks* because the sentence conveys the idea that lawyers are *ruthless*.

Two judges were involved in coding the responses. The first judge created the set of properties that reflected the interpretations written for each metaphor. The second judge then verified whether the property chosen was appropriate for the interpretation given. The second judge consulted the first judge when there were any disagreements and resolved any discrepancy. Once the set of properties had been decided, any property stated by a minimum of three participants was considered a salient property for that metaphor. Any properties stated by only two individuals were considered less salient properties. Properties stated only once were considered non-salient properties. This procedure allowed us to identify salient properties for all of the metaphors, but certain metaphors lacked less salient properties as there were no properties mentioned by at least two individuals. A list of the metaphors sorted by item group, accompanied by their salient and less salient properties, is presented in the Supplementary Material.

***Stimuli***. *Two* booklets were created for the metaphor interpretation task. One booklet listed half the original metaphors as similes (e.g., *Cities are like jungles* rather than *Cities are jungles*). In the second booklet, the topic-vehicle pairs were in the same order, but those items that were metaphors in booklet 1 were written as similes in booklet 2, and vice-versa.

#### Procedure

A researcher first administered the MoCA and MMSE, if the participant was a person diagnosed with AD, or only the MoCA if the participant was an elderly control. In addition, two subtests of the WAIS-IV were administered: similarities and the forward digit span task. Afterwards—or at another session if the previous tasks took longer than an hour—each participant was read either the first sentence of booklet 1 or 2 and asked to provide an interpretation of the sentence. For example, the first time the metaphor *Music is medicine* was read, the researcher asked the participant, *What is someone trying to say when he or she says that music is medicine?* If the participant could not provide an answer, or failed to mention a particular property, the researcher then asked the participant, *If someone were to say that music is medicine, what would they be trying to say about music?* This method helped prompt answers that reflected a particular property that could then be matched to the interpretation norms. After an interpretation had been given by the participant, and transcribed by the researcher, or if the participant was still unable to provide an adequate answer, the next sentence was read, and so on, for all 20 items. Participants were given unlimited time to provide an interpretation, and told it was fine if they could not think of an interpretation. Sessions involving the metaphor interpretation task lasted approximately 30 min.

### Results and discussion

Interpretation scoring followed a procedure similar to that used by Papagno (2001). A score of 2 was given if the interpretation mentioned a salient property, but a score of 1 if the interpretation mentioned a less-salient property. If the interpretation expressed a meaning completely different from the salient or less salient properties, or if the participant was unable to provide an answer, a score of 0 was given. Therefore, the maximum average interpretation score obtainable for a particular group was 2. In order to score the answers, one researcher first allocated a set of scores based on the transcriptions, while a second judge, who was blind to whether the interpretations came from a control or a person diagnosed with AD, also provided scores as a reliability check. The interclass correlation was 0.85 (*p* < 0.001). Because this reliability score was high, the scores from the first researcher were used in all subsequent analyses.

As mentioned above, two items were dropped from analysis because participants (AD patients and controls) repeatedly expressed difficulty providing an interpretation. Several participants expressed understanding the statement *Life is a journey*, but stated it was difficult to put into words a particular meaning. When interpretations were provided, most participants provided elaborate discussions about life in general rather than providing a particular property. The metaphor *Genes are blueprints* also caused confusion and was consequently dropped from analysis. For most participants, there was an initial period where the participant had to be told that the sentence meant genes “as in DNA” as opposed to *blue jeans*. Several participants (especially AD patients) expressed not understanding the concept *DNA*. Therefore, the metaphor interpretation scores for these items were not included in any of the analyses involving metaphor interpretation scores.

#### Group analyses

For AD patients, the mean metaphor score was 1.21 (*SD* = 0.49) and the mean simile score was 1.32 (*SD* = 0.19). For normal elderly controls, the mean metaphor score was 1.45 (*SD* = 0.14), and the mean simile score was 1.55 (*SD* = 0.31). Figure 1 in the supplementary materials displays these results. We first ran a repeated-measures ANOVA that compared AD patients' and elderly controls' general means for metaphor and simile interpretation scores. Group (AD vs. Control) was the between-subject factor and form (metaphor vs. simile) was the within-subject factor. The main effect of form was not significant [*F*_(1, 19)_ = 1.47, *p* = 0.24, η_*p*_ = 0.072], nor was the interaction [*F*_(1, 19)_ = 0.01, *p* = 0.95, η_*p*_ = 0.001]. These non-significant effects, in principle, go against the hypothesis that topic-vehicle words presented in metaphor form (*x is y*) would be harder to understand because they would need to be converted into simile form (*x is like y*), while similes allow for a direct comparison. Although the lack of a difference cannot rule out this hypothesis—in particular because it applies primarily to novel metaphors (e.g., Gentner and Bowdle, 2008)—it is important to note that the verbal interpretation task we employed requires a figurative interpretation of the relation between topic and vehicle, and thus metaphor and simile forms might yield the same interpretation strategy, with both leading to a figurative interpretation. Due to this lack of difference, we refer to these expressions simply as metaphors. The mean metaphor interpretation score for AD patients was 1.26 (*SD* = 0.29) vs. 1.50 (*SD* = 0.19) for normal controls, and this difference was significant when we ran the repeated-measures ANOVA [*F*_(1, 19)_ = 4.82, *p* < 0.05; η_*p*_ = 0.20]. We also compared this difference by items, and again found a significant difference [*t*_(17)_ = −2.17, *p* < 0.05, *r* = 0.47]. Thus, the difference between groups, regardless of expression type or other stimulus variables (see below), suggests an impairment in figurative language interpretation in AD, an effect that has not been obtained in tasks that require overt explanation of figurative meaning (e.g., Papagno, 2001; Amanzio et al., 2008). Recall, however, that those null differences were found for conventional metaphors only. We next examine how item variables influenced interpretation.

#### Effects of aptness and familiarity

We first examined whether the COCA counts would correlate with the Google counts in order to validate Internet frequencies as predictors of familiarity. We found a positive correlation between the Google search counts and the COCA counts [*r*_(16)_ = 0.49, *p* < 0.05], which suggests the Google counts collected using the Roncero et al. (2006) method tap into how familiar participants may be with a metaphorically expressed meaning. In order to better understand the aptness and familiarity effects on metaphor interpretation, we ran a multiple regression with the aptness ratings, Internet frequency counts, and COCA counts as predictors and AD patients' interpretation scores as the dependent variable. The overall model was significant [Adj *R*^2^ = 0.43, *F*_(3, 14)_ = 5.34, *p* < 0.05]. Among the individual predictors, however, aptness ratings alone were a significant predictor of interpretation scores (*t* = 3.58, *p* < 0.01), but not the Internet frequency counts (*t* = −0.23, *p* = 0.82) nor the COCA counts (*t* = −0.38, *p* = 0.71). See Figure 2 in the supplementary materials for a scatterplot between aptness ratings and AD patients' interpretation scores. Similar results were found for controls' interpretation scores. Again, the overall model was significant [Adj *R*^2^ = 0.43, *F*_(3, 14)_ = 5.27, *p* < 0.05], and aptness ratings were a significant predictor (*t* = 3.58, *p* < 0.01), but not Internet frequency counts (*t* = −0.09, *p* = 0.93), nor COCA counts (*t* = −1.06, *p* = 0.31).

#### Participant variables

The results are suggestive of a general aptness effect on metaphor interpretation—with overall better interpretation when the relation between topic and vehicle is deemed apt rather than familiar. However, other participant variables need to be taken into account before we can generalize over the effect of aptness on comprehension. In order to better understand the factors influencing performance on the different metaphor factors, we first compared participants' working memory as measured by the simple digit span task. No difference was found between AD patients (*M* = 11.18; *SD* = 2.44) and controls [*M* = 11.60; *SD* = 1.07; *t*_(14.01)_ = 0.52, *p* = 0.61, *r* = 0.14]. AD patients had a mean similarity judgment score of 17.18 (*SD* = 10.13), while controls' score was 26.20 (*SD* = 1.81). This difference was significant [*t*_(10.70)_ = −2.90, *p* < 0.05, *r* = 0.66]. Finally, AD patients' metaphor interpretation scores were found to correlate with similarity judgment scores [*r*_(9)_ = 0.68, *p* < 0.05], but not digit span scores [*r*_(9)_ = 0.06, *p* = 0.86]. Therefore, the ability to abstract a relationship between two objects might be considered a strong predictor of patients' abilities to interpret metaphor.

## Study 2: subjective familiarity ratings by elderly individuals

In our main study, aptness ratings provided by participants were strong predictors of metaphor comprehension. In contrast, two objectives measures of familiarity, Internet counts and COCA frequency counts, failed to predict which metaphors would be better interpreted by AD patients. This result is surprising considering studies (e.g., Amanzio et al., 2008) suggesting that familiarity is a strong predictor for the correct interpretation of metaphors. One crucial difference between our metaphors and those of Amanzio et al., however, is that while they used conventional metaphors (obtainable from the dictionary) as their set of familiar and easily understandable metaphors, we used metaphors whose meaning requires understanding a perceived relationship between a topic-vehicle pair even when that metaphor is unfamiliar (e.g., *Deserts are ovens)*. A valid concern, however, is that our measures may have more properly reflected *frequency* rather than *familiarity per se*. We assume that more frequent expressions are also more familiar, but such measures only indirectly reflect *familiarity* when personal experience itself is considered, and may exist on a more subjective level entirely. For example, one may only have heard a particular expression such as *Lawyers as sharks* a few times (say, less than ten), but nevertheless consider the expression rather familiar. Our concern in the first study is that such judgments are influenced by aptness. More specifically, apt metaphors are more easily understood (Chiappe et al., 2003a), and this ease of interpretation might lead people to have the impression that these metaphors are actually more familiar than they are (Thibodeau and Durgin, 2011; see also Jacoby and Whitehouse, 1989; Whittlesea and Williams, 2001, and Westerman, 2008 for parallels in visual recognition memory). These concerns motivated our preference for objective measures of familiarity in the study on metaphor interpretation, but it may have also come at the cost of only examining familiarity indirectly: frequency (occurrence across a medium), rather than familiarity, which would have predicted better interpretations.

In the present study, we examined the predictive value of subjective familiarity ratings. A group of elderly adults was asked to judge how familiar they found the expressions used in the metaphor interpretation study. To ensure that participants would not be biased by aptness—a central concern of our previous study's use of objective measures—participants were specifically told to ignore all aspects related to the metaphor, except their personal experience. We then examined if these subjective ratings of familiarity would predict AD interpretation scores that were collected in the previous study. Because our concern from the beginning of the investigation has been the bias effect of aptness on familiarity ratings, we predicted that there would be a significant relationship between aptness and familiarity ratings despite our best efforts to remove such bias. This result reflects the ease of interpretation effect, whereby people consider statements more familiar because they are more quickly or easily interpreted. Furthermore, in case the subjective familiarity ratings would be a significant predictor of interpretation scores, the aptness ratings collected in the previous study would nevertheless be found to be the stronger predictor of interpretation.

### Methods

#### Participants

Twenty elderly adults (age range: 67–88, 15 females) were recruited from a list of participants at the Bloomfield Center for Research in Aging (Lady Davis Institute, Jewish General Hospital, Montreal). These participants are accustomed to being recruited for various studies, have no known psychiatric illness nor signs of dementia, and all participants obtained scores over 26 (normal range) on a MoCA task that was administered before the metaphor familiarity task. On this occasion, participants were also administered a large battery of tasks for various unrelated studies, which included the present familiarity ratings. Participants were given monetary compensation for their time.

#### Familiarity ratings

Ratings were collected vocally. Participants were told they would be read a series of metaphors, and they were to rate how often they had heard the expression in the past, employing a scale ranging from 1 (not at all) to 7 (practically every day). They were further told that they were not to rate how well they liked the expression, nor how well they understood it, but to focus solely on how often they had heard this given expression previously. Finally, they were told that while some expressions may be quite familiar, others could be ones they had never heard before, case in which they should simply answer honestly with a rating of 1. After the participant confirmed understanding the instructions, the researcher then read each metaphor in the following manner: *From 1 to 7, 1 being not at all, how often have you heard the metaphor…* (e.g. *Music is medicine)?* All participants were read each expression in this manner one-by-one, and all participants were presented the expressions in the same order. The participant then stated a number between 1 and 7 as their rating, and the researcher recorded this response. If the participant chose to change their rating before moving to the next expression, this new rating replaced the first one. When participants gave two responses (e.g., stating *“I'd give that a 2 or a 3*”), the lower number was always chosen.

### Results and discussion

The collected familiarity ratings are presented in the Supplementary Material. The mean metaphor rating was 3.45 (*SD* = 1.84). These ratings correlated significantly with both the Internet counts [*r*_(16)_ = 0.61, *p* < 0.01] as well as the COCA counts [*r*_(16)_ = 0.59, *p* < 0.01], which suggests that participants' ratings were tapping into the general frequency of the expression. However, as predicted, subjective familiarity ratings were also significantly correlated with the aptness ratings obtained in the previous study [*r*_(16)_ = 0.70, *p* < 0.01], which suggests that ratings were affected by an items' aptness level. Indeed, the significant correlation with aptness may explain why these subjective measures of familiarity, unlike the previously collected objective measures, were a significant predictor for the interpretation scores of AD patients from study 1 [*r*_(16)_ = 0.53, *p* < 0.05]. Noting these three significant relationships (aptness and familiarity, aptness with interpretation scores, familiarity with interpretation scores), we checked for mediation by running a regression with both aptness and familiarity ratings as predictors, and AD patients' interpretation scores as the dependent variable. The overall model was significant [*F*_(2, 15)_ = 8.26, *p* < 0.01], but among the individual predictors, the only one significant was aptness (*t* = 2.78, *p* < 0.05; familiarity, *t* = 0.16, *p* = 0.88).

The results thus allow us to argue that AD patients' superior interpretation of metaphors rated more familiar is fully mediated by the aptness level of these metaphors. Furthermore, the semi-partial correlation between AD patients' interpretation scores and aptness ratings was 0.50, but only 0.03 for familiarity ratings; a large drop from 0.53 when familiarity ratings are considered alone. Therefore, the results cement our findings from the previous study: when considering the types of metaphors that will be best interpreted by AD patients, aptness rather than familiarity is a more important predictor. Metaphors that older adults consider more familiar will be better interpreted by AD patients, but this relationship depends on the aptness level of these metaphors. In other words, metaphors considered more familiar are better understood by AD patients because they are also inherently more apt.

## General discussion

Thus far, only studies employing a multiple choice or a match-to-target kind of test have found deterioration in figurative language comprehension in mild-to-moderate AD patients (e.g., Chapman et al., 1997; Rassiga et al., 2009). A similar pattern was also obtained by two of the other four studies investigating metaphor comprehension in AD (Winner and Gardner, 1977; Maki et al., 2012). One problem with such studies is that the task provides a literal interpretation together with the figurative one; in those circumstances, AD patients may have difficulty inhibiting the literal interpretation in order to select the figurative one. When tasks require free verbal interpretations of familiar figurative language, AD patients usually do not differ from controls (e.g., Papagno, 2001; Amanzio et al., 2008). In the present study, participants were asked to provide interpretations for different metaphors and similes, and while metaphors and similes did not differ within groups, AD patients produced overall worse figurative interpretations than did controls. Crucially, however, we also show that the pattern of impairment in AD depends both on the patient's abstraction abilities and on the aptness of the metaphor. In this section we focus on two key issues related to the pattern of figurative language performance in AD: stimulus variables, in particular aptness and familiarity, and whether the meaning of a figurative expression is constructed or stored in memory. We follow this discussion with a proposal for how aptness and abstraction abilities interact to yield metaphor interpretation.

### Aptness and familiarity

Familiarity with a figurative expression has been one of the most studied variables investigated in both, the psycholinguistics and the cognitive neuropsychology literatures (see Gibbs, 2008; Rapp and Wild, 2011). The studies by Papagno (2001) and Amanzio et al. (2008), more specifically, had both employed familiar (dictionary listed) metaphors; and in both studies, patients had no difficulty with familiar metaphors, on the assumption that their meanings could be accessed from memory. Amanzio et al. showed moreover that the patients had greater difficulty interpreting novel metaphors—on the assumption that they would need to compute a novel meaning and this ability might be affected in AD. From these results, Amanzio et al. argued that novelty was a crucial variable for predicting performance. In the present study, we showed the importance of *aptness* for predicting metaphor interpretation. Patients' interpretations, despite being worse than the controls', were particularly affected by item variables. Furthermore, when we examined aptness and familiarity as predictors of interpretation scores, the pattern of results point to aptness playing a bigger role than familiarity for metaphors whose meanings require the computation of a relationship between the topic and vehicle. Finally, even when we did find a significant relationship between subjective ratings of familiarity and metaphor interpretation, this relationship was found to be fully mediated by aptness. Therefore, a familiar metaphor is understood well because it is apt.

In the world of music, a cliché question is whether a song is popular because it is good, or good because it is popular. This question has an analogous one within the world of metaphor: is a metaphor apt because it is familiar, or familiar because it is apt? (Thibodeau and Durgin, 2011). In the present study, we were unable to identify a familiar metaphor that was not considered apt, despite working initially with a cohort of 84 metaphors (see Roncero and de Almeida, 2014, for the full set and norms). In contrast, it was possible to find metaphors considered apt but not familiar. We believe this reflects a tendency, perhaps a necessity, for expressions to be apt before they are familiar because aptness more so than familiarity will breed comprehensibility. Indeed, consider an extremely inapt statement such as *Flags are dust*. One could recite this metaphor *ad nauseum* and probably never compose a meaning other than the seemingly anomalous literal one. Thus, some level of aptness is needed to give metaphors a comprehensible “lift-off” (Chiappe et al., 2003b; Roncero et al., 2006). Consistent with this idea, we found that the relationship between subjective (i.e., rated) familiarity and interpretation ability was fully mediated by aptness in the study on metaphor interpretation. Note also that while Amanzio et al. (2008) stressed the importance of item familiarity for predicting AD patients' interpretation of metaphors, the metaphors they used were also highly apt. Furthermore, it is unclear whether the AD patients in that study had significantly lower or similar abstraction abilities than the control participants, which we have found to be a key predictive variable for AD patients' interpretation of metaphors.

The results also remind us of a basic characteristic of language: comprehension is possible for statements that have never been heard before because language is compositional and systematic, and thus productive. Novel expressions (or novel combinations of familiar words) such as metaphors are understood because they hinge on our ability to compose meanings and thus evaluate the relationship between the expressions' constituents even when they are not familiar. Consider, for example, the metaphor *Deserts are ovens*. Participants consistently stated they had rarely to never heard this expression before, an expression that had low COCA and Internet counts, but one which AD patients and normal participants almost always correctly stated its metaphorical meaning. Familiarity is probably more important for opaque relationships, i.e., those that are not deducible from the words in the expression as is the case of idioms where the meaning of the expression and its constituent lexical items seem to share an arbitrary relationship. In contrast, in a metaphor where the vehicle used is often selected to express a particular relationship (e.g., *Juliet is the sun*), the aptness of the expression and the abstraction abilities of the individual can be expected to play a greater role in determining how easily it is interpreted correctly. In summary, while familiarity plays a role in metaphor comprehension, as shown in previous studies, the aptness of a metaphor seems to play yet a greater role.

### Abstraction, retrieval, and construction

These variables cut across another key distinction in how the meaning of a metaphor is attained: whether it is by accessing a stored representation in memory or whether it is constructed online. As our data suggest, being able to take advantage of the qualities related to aptness depends on the extent to which the ability to abstract is preserved. If so, patients with more impaired abstraction abilities can be expected to demonstrate more difficulty constructing interpretations for metaphors. If the level of abstraction required is low, however, it is possible that even patients with more impaired abstraction abilities can demonstrate normal levels of comprehension. For example, it is easier to determine the relationship between carrots and broccoli (both are vegetables) than between music and tides (both have rhythms). In our data, we noticed that among the best-interpreted metaphors by AD patients was *Deserts are ovens*, where the salient property was *hot*; and also a majority of the AD patients correctly interpreted the metaphor *Hair is a rainbow* as meaning hair is *colorful*. However, neither of these metaphors had been rated apt, and both had low Internet frequency counts (*n* < 5). These findings support the argument that metaphors can be easily interpreted, despite not being very apt, when the abstraction level demanded is low, and perhaps especially true when the properties needed for interpretation are concrete and sensory in nature (Aisenman, 1999). In contrast, a metaphor such as *Families are fortresses*, which would be more difficult for people with low abstraction ability, makes no reference to a specific literal property of fortresses (e.g., made of stone), and refers to more abstract concepts of security and protection. These observations support one of our main suggestions: insofar as different metaphors require constructing meanings that reflect different abstraction levels, individuals who are better abstractors should have an easier time interpreting metaphors. Although this suggestion is made within the limited scope of our investigation with Alzheimer's patients, it points to an important aspect of metaphor—and figurative language—interpretation in general: the ability to infer intended messages from the often anomalous linguistic expressions requires a computational mechanism capable of generating properties and relations beyond linguistic denotation. We suggest that this mechanism is intrinsically associated with comprehenders' abstracting capacities.

The pattern of our results then supports the idea that abstraction plays a key role in building figurative representations, with the aptness of an expression being the most important stimulus property. Whereas Amanzio et al. (2008) argued that novelty matters, we would argue that aptness matters more. Statements are familiar because they are apt, and unfamiliar statements should be understood by AD patients when the aptness level is sufficiently high. Also, while it is difficult to compare our study to that by Amanzio et al.'s, given the numerous methodological differences (language, materials, task, and participant variables), a critical issue seems to be the metaphors used in both studies. Amanzio et al. employed conventional Italian metaphors whose meanings were retrievable from the dictionary, and often included topics that were simply proper names (e.g., *Marco is a lion*). Metaphors that use proper names as topics only require selecting a property of the vehicle; and this process is more consistent with the need to simply retrieve an associated property (perhaps a typical property of lions) to use in the predication. In contrast, the familiar metaphors used in the present study never used topics that were proper names; thus, even for familiar metaphors, patients needed to construct a particular relationship between the topic and vehicle to attain an interpretation. With regards to the computations required for interpretation, then, our set of metaphors could be taken as similar to the novel metaphors used by Amanzio et al.: rather than being retrieved from memory, their meanings had to be constructed, based on the properties triggered by the vehicle that could be predicated of the topic. This process can be expected to be easier for all participants when the statements are apt.

Finally, these results also reinforce a key contrast between (“unfrozen”) metaphors and other (“frozen”) figurative language types. Whereas patients can rely more on retrieval processes for familiar expressions that have a specific associated meaning, many common metaphors require patients to deploy abilities that allow them to construct an appropriate interpretation. As we have seen, most studies on proverbs, idioms, sarcasm, and metaphor suggest that AD patients' primary difficulty with figurative language seems to be the ability to inhibit a literal interpretation when it is available (e.g., in multiple-choice tasks). But when there is no competition from a literal interpretation and when expressions are familiar, AD patients often did not differ significantly from healthy controls. For less familiar expressions, however, AD patients had shown a reduced ability compared to controls, suggesting an impaired ability to build new meanings. The present study found, however, that the ability to build new meanings is associated to the capacity to abstract away from literal meaning (compute related predicates) and the aptness of the expression.

### Interpreting metaphors in alzheimer's

While we have shown evidence for the role of both, abstraction as a participant variable and aptness as a stimulus variable, in metaphor comprehension, it is not clear yet how the two interact to yield a successful interpretation. As measured by the WAIS-IV similarities subtest, abstraction is a process required to go beyond word meanings in search of properties that can account for how two referents might be related. While any two referents can be related (e.g., two things that are concrete or co-exist on Earth), finding appropriate relations requires an examination of which properties *P* can be predicated of any two given objects *x* and *y* such that *P*(*x*) and *P*(*y*) can be deemed true. Aptness is the variable that facilitates this process in metaphor comprehension. An apt metaphor is one in which sets of properties about the vehicle can be attributed to the salient properties of the topic. The process of finding which properties of the vehicle can be predicated about the topic, relies on accessing sets of predicates in memory (e.g., [*ruthless*[*shark*]], [*carnivore*[*shark*]) or building them anew ([*sneaky*[*shark*]]) and applying them to the topic ([*ruthless*[*lawyer*]], [*sneaky*[*lawyer*]]) to yield an interpretation of the metaphor. Furthermore, this process can interact with the progression of AD. Over the course of the disease, as semantic memory deteriorates progressively (Chertkow and Bub, 1990; Mårdh et al., 2013) less familiar information is expected to be “lost” first (Laisney et al., 2011) and semantic categories are expected to become increasingly prototypical over time (Chertkow and Bub, 1990; Laisney et al., 2011; Mårdh et al., 2013). Therefore, metaphors whose interpretations rely on less salient properties of topic-vehicle relations can be expected to engender greater difficulty. Apt metaphors, in contrast, often rely on vehicles whose properties can be easily predicated to the topic (Glucksberg, 2003; Jones and Estes, 2006) and may remain comprehensible for AD patients because their properties might still be available in semantic memory.

We would thus argue that patients who are better abstractors (i.e., closer to or at normal abstraction ability) can interpret even unfamiliar metaphors when these are apt. We suggest that they are capable of doing so because they can compute not only the literal meanings of words but also search for the vehicle predicates that make a predication about the topic appropriate. It is also worth noting that the interpretation errors made by AD patients in the metaphor interpretation study were either geared toward an alternative interpretation—neither compatible with the metaphor nor literal—or simply revealed an inability to produce an interpretation, thus replicating Papagno's (2001) results. These results suggest that patients recognized that the expressions presented to them were false if interpreted literally, but may have lost the ability to search for the predicates that would enable them to construct an alternative interpretation. For example, patients who were unable to interpret a metaphor would typically respond with statements such as “that makes no sense, alcohol can't be a crutch!” Therefore, these patients have retained an ability to recognize whether a statement is true of the world (i.e., that metaphors are literally false), but have difficulty making the correct abstraction.

## Conclusion

Davidson (1978) had proposed that metaphors invite us to appreciate some fact rather than expressing it overtly. Although families are not literally fortresses, a metaphor does call our attention to what fortresses can possibly predicate of families. Overall, our study suggests that the capacity to appreciate a metaphor is available to patients with AD when they can perform abstractions—thus to go beyond literal meaning—when such metaphors have a sufficient level of aptness.

### Conflict of interest statement

The authors declare that the research was conducted in the absence of any commercial or financial relationships that could be construed as a potential conflict of interest.

## Footnotes

^1^We use “meaning” in a loose sense often to cover both a literal meaning (roughly, what is said) and the intended message or even what is implicated by an expression (e.g., “metaphor meaning”), which might differ substantially from what is literally expressed. When necessary we make adjustments to our use of “meaning” to reflect these distinctions.

^2^We should also note that of the 22 studies on figurative language in AD—the 20 reviewed by Rapp and Wild (2011) and two more recent ones (Yamaguchi et al., 2011; Maki et al., 2012)—several have employed figurative language as a diagnostic tool for early dementia (e.g., Code and Lodge, 1987; Santos et al., 2009; Yamaguchi et al., 2011), while others have actually investigated the nature of figurative language understanding obtained from the pattern of linguistic and cognitive deficits in AD. We restrict our discussion to the latter types, which are more closely related to the present study.

^3^We will use “semantic composition” or “compositional” to refer to expressions from which a meaning is obtained by computing the (denotational) meaning of the constituent words and how they combine syntactically. How a non-compositional meaning is obtained (i.e., a figurative meaning not based on the words stated) remains a matter of debate. Some have argued that certain idioms, like *kicked the bucket*, are expressions that behave as if they are lexicalized (Swinney and Cutler, 1979; but see Holsinger and Kaiser, 2013) or simply accessed in memory (McCabe, 1988; Caillies and Declarcq, 2011). Meanwhile, others (e.g., Searle, 1979) have argued that obtaining the figurative meaning of an expression requires one to first entertain its literal meaning to later discard it, or that a figurative meaning can actually be obtained “directly” (Gibbs, 2001; Glucksberg, 2003). These debates, however, will not be resolved in the present paper.

^4^Although we distinguish figurative expressions according the frozen/non-frozen contrast, what is important is how the intended message conveyed by these expressions is attained—whether it is stored or computed anew.

^5^ We also note that Maki et al. (2012) reported worse interpretation for conventional Japanese metaphors (single-word and copular). We would argue, however, that their use of a multiple-choice task could have again biased patients to select the literal foil.
