# Supplementary material for: The importance of being apt: metaphor comprehension in Alzheimer's disease
Source: Front Hum Neurosci. 2014 Dec 2;8:973. doi: 10.3389/fnhum.2014.00973 (PMC4251318; doi:10.3389/fnhum.2014.00973)

| Supplementary Material | | | | | | | | |  |  |
| --- | --- | --- | --- | --- | --- | --- | --- | --- | --- | --- |
| Stimuli employed in the study, organized according to the Aptness and Familiarity scales. Aptness ratings were obtained by an independent group of elderly individuals (*N*=20) who did not participate in the experiment. Familiarity was obtained from Internet counts following the method described in Roncero et al. (2006). Salient and Less Salient properties were given by another group of healthy elderly individuals (*N*=20) who did not participate in the experiment. Expressions in the metaphor form (*x is y*) are shown. The study also included the same topic (*x*) and vehicle (*y*) pairs in simile form (*x is like y*). See details in the main text. | | | | | | | | |  |  |
|  |  |  |  |  |  |  |  |  |  |  |
|  |  |  |  |  |  |  |  |  |  |  |
|  |  |  |  |  |  |  |  |  |  |  |
| Expression |  | Aptness |  | Familiarity | | | Salient Property | Less Salient Property |  | |
|  |  |  |  | Google | CoCA | Subjective |  |  |  |  |
| Alcohol is a crutch |  | 5.81 |  | 28 | 2 | 4.65 | relied upon, addictive | numbing |  |  |
| Anger is fire |  | 5.77 |  | 30 | 16 | 1.40 | uncontrollable, destructive |  |  |  |
| Anger is a heart |  | 1.82 |  | 0 | 3 | 1.05 | emotional | dangerous |  |  |
| Cigarettes are time bombs |  | 5.86 |  | 0 | 0 | 3.75 | Death, dangerous |  |  |  |
| Cities are jungles |  | 4.86 |  | 17 | 4 | 5.20 | crowded | dangerous |  |  |
| Deserts are ovens |  | 2.85 |  | 0 | 1 | 1.75 | hot |  |  |  |
| Education is a stairway |  | 6.36 |  | 1 | 0 | 4.30 | betterment | eternal |  |  |
| Exams are hurdles |  | 5.54 |  | 4 | 5 | 3.15 | challenging, allows progress |  |  |  |
| Families are fortresses |  | 4.81 |  | 2 | 0 | 2.90 | strong, protective | safe, united |  |  |
| Hair is a rainbow |  | 2.62 |  | 2 | 5 | 1.00 | colourful | crown, beautiful |  |  |
| Knowledge is light |  | 5.82 |  | 30 | 22 | 2.40 | enlightening | bright |  |  |
| Knowledge is power |  | 6.52 |  | 30 | 193 | 6.45 | empowering |  |  |  |
| Lawyers are sharks |  | 4.95 |  | 30 | 4 | 5.65 | ruthless | sharp |  |  |
| Life is a bottle |  | 2.86 |  | 2 | 0 | 1.10 | encased | empty or full |  |  |
| The mall is a zoo |  | 5.45 |  | 4 | 0 | 3.55 | chaotic, dangerous | crowded |  |  |
| Men are fish |  | 1.61 |  | 2 | 0 | 1.85 | catchable | slippery, gullible |  |  |
| Music is medicine |  | 5.86 |  | 30 | 5 | 4.40 | soothing, enjoyable | healing |  |  |
| Time is money |  | 5.72 |  | 30 | 133 | 6.60 | valuable | limited, wasted |  |  |


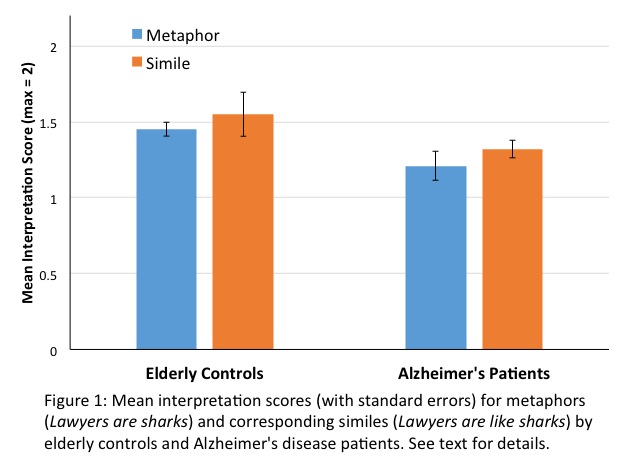

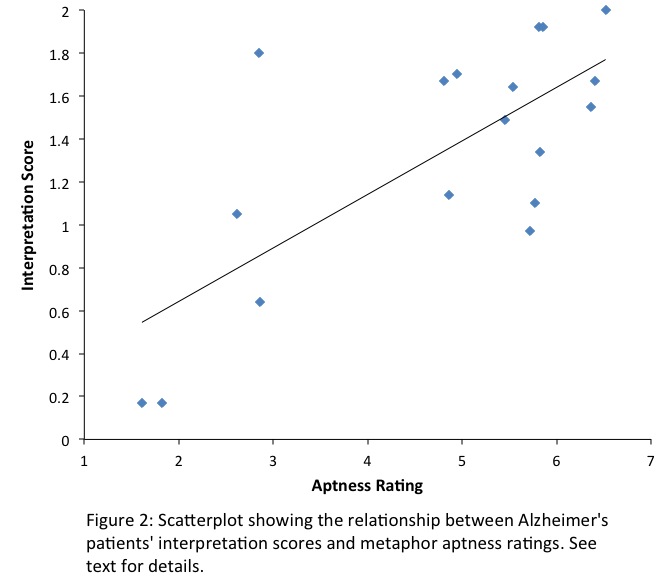

Supplement: Supplementary file 1 [file DataSheet1.DOCX]
